# Microbial changes resulting from VSG attenuate MASLD by modulating bile acid metabolism and the intestinal FXR-FGF19 axis

**DOI:** 10.1128/msystems.00634-25

**Published:** 2025-10-20

**Authors:** Yi Xia, Jinpu Yang, Shixian Lu, Weixin Cheng, Mengting Ren, Zhening Liu, Ling Yang, Qien Shen, Yujie Liang, Hangkai Huang, Minjie Chen, Xinxin Zhou, Mosang Yu, Feng Ji, Chengfu Xu

**Affiliations:** 1 Department of Gastroenterology, The First Affiliated Hospital, Zhejiang University School of Medicine 26441, Hangzhou, China; 2 Department of Gastroenterology, Affiliated Hangzhou First People's Hospital, School of Medicine, Westlake University, Hangzhou, China; 3 Department of Gastroenterology, Shengzhou Hospital of Traditional Chinese Medicine, Shaoxing, China; 4 Cancer Center, Department of Gastroenterology, Zhejiang Provincial People's Hospital (Affiliated People's Hospital), Hangzhou Medical College 74678, Hangzhou, China; University of Illinois Chicago, Chicago, Illinois, USA

**Keywords:** vertical sleeve gastrectomy, MASLD, gut microbiota, bile acid, FGF19

## Abstract

Vertical sleeve gastrectomy (VSG) is a highly effective intervention for metabolic dysfunction-associated steatotic liver disease (MASLD) and is associated with significant alterations in the gut microbiota. However, the precise mechanisms underlying its metabolic benefits remain poorly understood. In this study, we revealed that VSG mitigates MASLD by reshaping gut microbiota-mediated bile acid metabolism. Through integrated 16S rRNA sequencing, targeted metabolomics, and functional validation experiments, we demonstrated that VSG markedly enhances bile salt hydrolase (BSH) activity within the gut microbiota, resulting in elevated levels of unconjugated bile acids. These unconjugated bile acids serve as potent agonists for the intestinal farnesoid X receptor (FXR), thereby activating the intestinal FXR-fibroblast growth factor 19 signaling pathway. This activation leads to significant improvements in metabolic health, including enhanced glucose regulation and attenuated hepatic lipid accumulation. Fecal microbiota transplantation (FMT) from VSG-treated rats replicated these metabolic improvements, whereas antibiotic treatment abolished these beneficial effects, highlighting the indispensable role of the gut microbiota in mediating the anti-MASLD effects of VSG. Importantly, inhibition of intestinal FXR signaling negated the metabolic benefits of FMT, further emphasizing the critical role of the gut microbiota-BSH-FXR axis. Our findings reveal a novel mechanism by which VSG alleviates MASLD through gut microbiota-dependent activation of intestinal FXR, offering new perspectives for microbiome-targeted therapeutic strategies in MASLD.

**IMPORTANCE:**

Fecal transplantation from bariatric surgery patients and mice to germ-free mice has shown that the gut microbiota may contribute to metabolic benefits after bariatric surgery. However, the mechanisms by which the gut microbiota contributes to metabolic benefits after bariatric surgery require further investigation. To address this gap, we investigated the effects of the vertical sleeve gastrectomy (VSG) gut microbiota on metabolic dysfunction-associated steatotic liver disease (MASLD) *in vivo* and elucidated its underlying mechanisms. Our study demonstrated that VSG significantly improved the gut microbiota, especially by increasing bile salt hydrolase (BSH) activity, in MASLD rats. Increased BSH activity significantly increased the proportion of FXR-agonistic bile acids and further activated the intestinal FXR-FGF19 axis, thereby improving MASLD. These findings explored the key roles and mechanisms of the gut microbiota in the metabolic benefits of VSG, offering new microbiome-based treatment strategies.

## INTRODUCTION

Metabolic dysfunction-associated steatotic liver disease (MASLD) encompasses a spectrum of liver conditions ranging from simple hepatic steatosis to metabolic-associated steatohepatitis (MASH) (1, 2). These progressive conditions can evolve into more severe liver diseases, such as liver cirrhosis and hepatocellular carcinoma. Approximately 30% of individuals worldwide are affected by MASLD, which imposes a significant socioeconomic burden (3). Therefore, effective strategies for its prevention and treatment are urgently needed to reduce the disease burden associated with MASLD.

Bariatric surgery (BS) is currently the most effective method for achieving long-term weight reduction and alleviating MASLD (4). Common bariatric surgeries include vertical sleeve gastrectomy (VSG) and Roux-en-Y gastric bypass. A recent study revealed that a greater percentage of patients achieved remission of obesity-related MASH after bariatric surgery than after lifestyle interventions and optimal medical therapy (5). However, its invasiveness and irreversibility limit the choice of BS. Therefore, the transformation of bariatric surgical intervention into a more efficient and less invasive treatment for MASLD remains an important challenge.

The gut microbiota contributes significantly to the development of MASLD (6). Compared with those of healthy individuals, the gut microbiota of MASLD individuals differs, and species richness greatly decreases in MASLD patients (7). Interestingly, bariatric surgery has been found to improve gut microbial dysbiosis in patients with MASLD and is significantly correlated with its metabolic benefits (8). Fecal transplantation from bariatric surgery patients and mice to germ-free mice has shown that the gut microbiota may contribute to metabolic benefits after bariatric surgery (9). However, the mechanisms by which the gut microbiota contributes to metabolic benefits after BS require further investigation.

In this study, we revealed that VSG alleviates MASLD by modulating gut microbiota-mediated bile acid metabolism. VSG enhances bile salt hydrolase (BSH) activity, increasing unconjugated bile acids that activate intestinal farnesoid X receptor (FXR) signaling, leading to improved metabolic health. Fecal microbiota transplantation (FMT) from VSG-treated rats replicated these benefits, whereas antibiotic treatment or FXR inhibition abolished them, underscoring the gut microbiota-BSH-FXR axis as a key mechanism. Our findings highlight the therapeutic potential of targeting this axis in MASLD treatment.

## RESULTS

### VSG restores metabolic health and modulates lipid and glucose metabolism in high-fat diet (HFD)-induced MASLD rats

**Fig 1:**
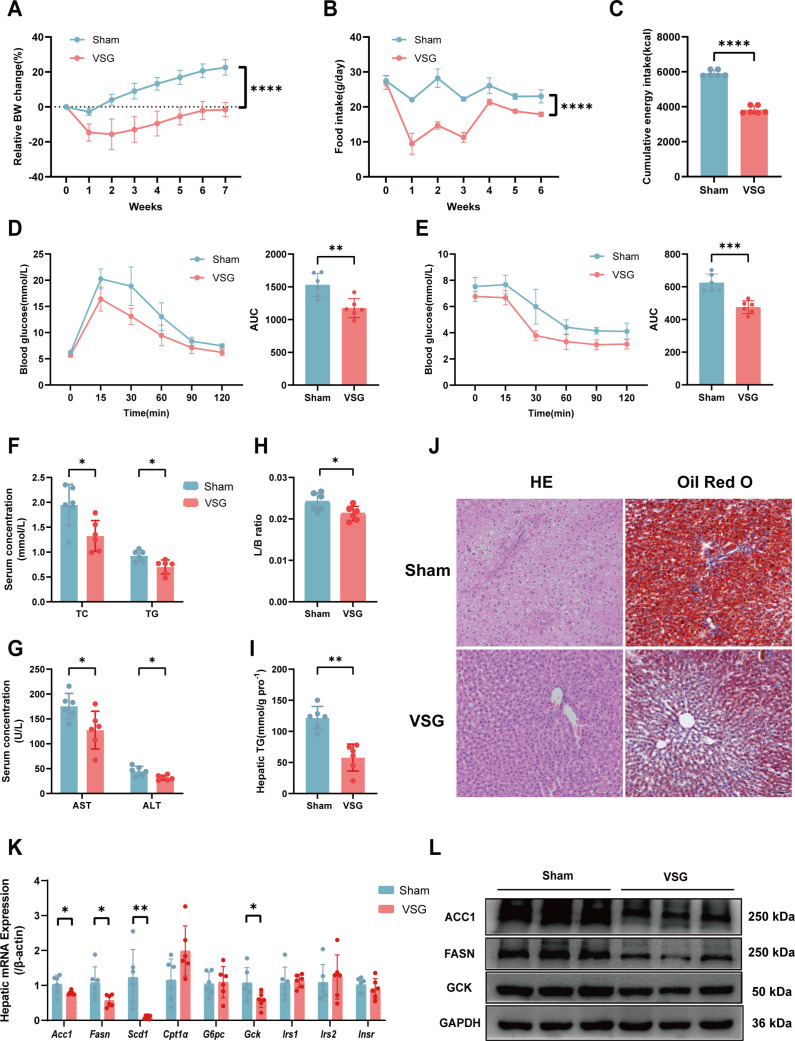
VSG restores metabolic health and modulates lipid and glucose metabolism in HFD-induced MASLD rats. (**A**) Relative body weight change. (**B**) Food intake. (**C**) Cumulative energy intake after VSG or sham surgery. (**D**) Glucose tolerance test with the corresponding area under the curve (AUC). (**E**) Insulin tolerance test with the corresponding AUCs. (**F**) Serum TC and TG levels. (**G**) Serum AST and ALT levels. (**H**) Liver:body weight ratio. (**I**) Liver TG contents at 7 weeks post-surgery. (**J**) Representative images of H&E staining and Oil Red O staining of rat liver tissues. (**K**) Relative mRNA expression of genes related to lipid metabolism (*Acc1*, *Fasn*, *Scd1*, and *Cpt1α*) and glucose metabolism (*G6pc*, *Gck*, *Irs1*, *Irs2*, *and Insr*). (**L**) Representative Western blot analyses of proteins related to lipid metabolism (ACC1, FASN) and glucose metabolism (GCK). *n* = 6 individuals per group. Each point represents an individual rat. The data are presented as the means ± SDs. *P < 0.05, **P < 0.01, ***P < 0.001, ****P < 0.0001. The data were analyzed via unpaired Student’s *t*-test. ACC1, acetyl-CoA carboxylase 1; FASN, fatty acid synthase.

After 8 weeks of HFD feeding, a rat model of MASLD was successfully established (Fig. S1). To verify the influence of VSG on MASLD, we performed VSG in HFD-induced MASLD rats (9). Compared with sham control rats, VSG rats presented greater reductions in body weight and food intake (Fig. 1A through C). VSG rats also exhibited ameliorated glucose and insulin intolerance (Fig. 1D and E) and significantly decreased serum total triglyceride (TG), total cholesterol (TC), alanine aminotransferase (ALT), and aspartate aminotransferase (AST) levels (Fig. 1F and G). The liver-to-body weight ratio and accumulation of hepatic TG were also significantly decreased in the VSG group (Fig. 1H and I). Hematoxylin and eosin (H&E) and Oil Red O staining revealed that VSG markedly alleviated HFD-induced hepatic steatosis (Fig. 1J). These findings suggest that VSG alleviates HFD-induced MASLD in rats.

To further investigate the mechanisms by which VSG enhances lipid metabolism, we conducted western blotting and quantitative reverse transcription-PCR (qRT-PCR) to detect the key molecules involved in lipid synthesis and fatty acid β-oxidation pathways. VSG treatment downregulated the mRNA levels of acetyl-CoA carboxylase 1 (ACC1), fatty acid synthase (FASN), and stearoyl-CoA desaturase 1 (SCD1) (Fig. 1K). The protein expression levels of ACC1 and FASN also significantly decreased after VSG (Fig. 1L), indicating that VSG inhibited lipid synthesis. However, the mRNA level of carnitine palmitoyl transferase 1α did not change substantially after VSG (Fig. 1K). Moreover, VSG markedly downregulated the mRNA and protein levels of glucokinase (Fig. 1K and L). These findings suggest that VSG regulates hepatic lipid accumulation by inhibiting lipid synthesis and improving glucose metabolism by decreasing glucokinase levels.

**Fig 2:**
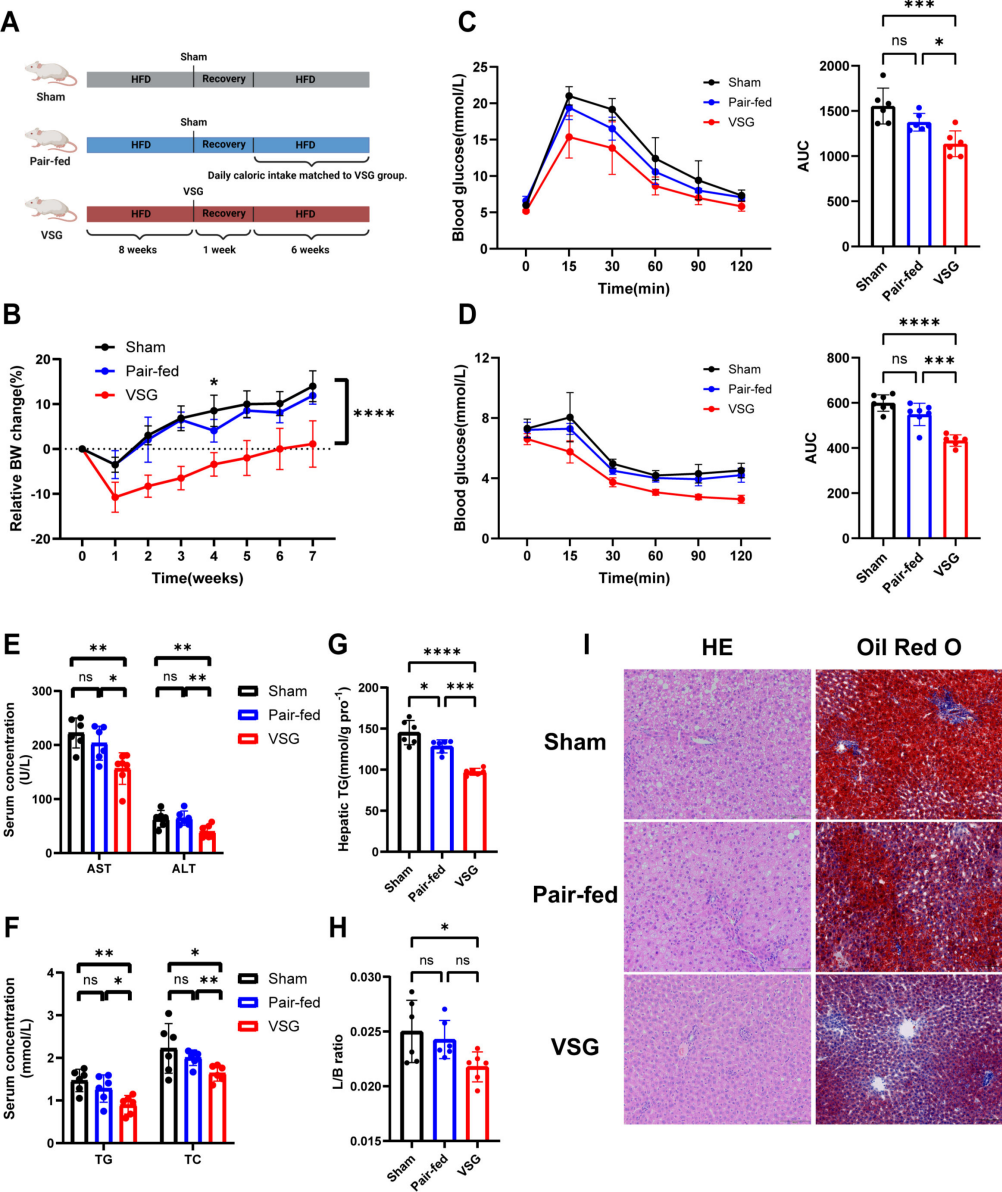
Paired feeding failed to replicate the metabolic benefits of VSG. (**A**) Illustration of the pair-feeding intervention experiment. (**B**) Relative body weight change. (**C**) Glucose tolerance tests with the corresponding areas under the curve (AUCs). (**D**) Insulin tolerance test with the corresponding AUCs. (**E**) Serum AST and ALT levels. (**F**) Serum TC and TG levels. (**G**) Liver TG contents and (**H**) liver:body weight ratios at 7 weeks post-surgery. (**I**) Representative images of H&E staining and Oil Red O staining of rat liver tissues. *n* = 6 individuals per group. Each point represents an individual rat. The data are presented as the means ± SDs. *P < 0.05, **P < 0.01, ***P < 0.001, ****P < 0.0001. The data were analyzed via one-way analysis of variance.

To determine whether the amelioration of MASLD by VSG was attributable to calorie restriction, a pair-feeding experiment was conducted (Fig. 2A). The results revealed that pair-feeding, although effective in reducing caloric intake, failed to replicate the comprehensive metabolic and hepatic improvements observed with VSG. Specifically, compared with the pair-fed group, the VSG group demonstrated superior efficacy in reducing body weight (Fig. 2B) and enhancing glucose metabolism (Fig. 2C and D). Additionally, compared with the pair-fed group, the VSG group presented significantly lower serum TG, TC, ALT, and AST levels in MASLD rats (Fig. 2E and F). Moreover, the reduction in hepatic lipid accumulation was substantially more pronounced in the VSG group than in the pair-fed group (Fig. 2G through I). These findings underscore that the therapeutic benefits of VSG in MASLD extend beyond simple calorie restriction, suggesting the involvement of additional mechanisms.

### The gut microbiota is critical for the metabolic benefit of VSG

**Fig 3:**
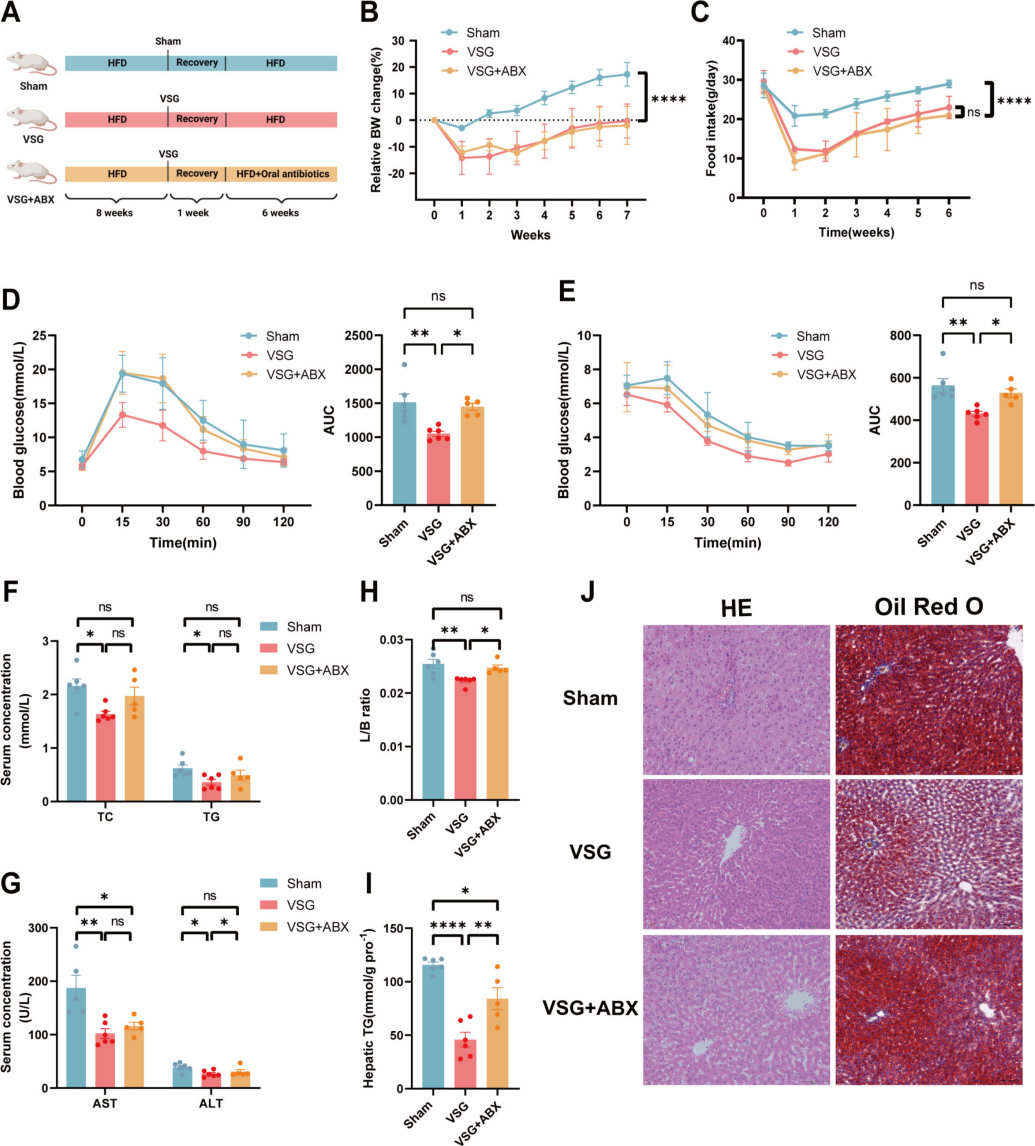
Microbiota depletion impaired metabolic improvements resulting from VSG surgery in HFD‐induced MASLD. (**A**) Illustration of the ABX treatment experiment. Diagram created via BioRender. (**B**) Relative body weight change. (**C**) Food intake. (**D**) Glucose tolerance test with the corresponding areas under the curve (AUCs). (**E**) Insulin tolerance test with the corresponding AUCs. (**F**) Serum TC and TG levels. (**G**) Serum AST and ALT levels. (**H**) Liver:body weight ratio. (**I**) Liver TG contents at 7 weeks post-surgery. (**J**) Representative images of H&E staining and Oil Red O staining of rat liver tissues. *n* = 5–6 individuals per group. Each point represents an individual rat. The data are presented as the means ± SDs. *P < 0.05, **P < 0.01, ***P < 0.001, ****P < 0.0001. The data were analyzed via one-way analysis of variance.

Considering that the therapeutic effects of VSG are not solely mediated by caloric restriction, we performed a comprehensive investigation to determine whether alterations in the gut microbiota contribute to the postoperative efficacy of VSG. First, we depleted the gut microbiota of the rats via an antibiotic mixture (ABX) (Fig. 3A). Although ABX treatment did not abrogate the differences in body weight or food intake between the rats in the sham and VSG groups (Fig. 3B and C), it substantially attenuated the beneficial effect of VSG on glucose and insulin tolerance (Fig. 3D and E). Moreover, ABX treatment abolished the reduction in the serum ALT level caused by VSG but had no effect on the serum TG, TC, or AST levels (Fig. 3F and G). The liver-to-body weight ratio and accumulation of hepatic TG were also significantly increased in the antibiotic-treated VSG group (Fig. 3H and I). H&E and Oil Red O staining revealed similar results (Fig. 3J). To rule out the potential direct effects of antibiotics (ABXs) on systemic metabolism in rats, we examined ABX intervention in SCD-fed rats. We found that ABX had no effect on body weight, food intake, glucose control, or hepatic steatosis in SCD-fed rats (Fig. S2). Furthermore, these results support our conclusion that elimination of the VSG-associated gut microbiota partially attenuates the metabolic benefits of VSG, reinforcing the role of the gut microbiota in mediating these effects.

**Fig 4:**
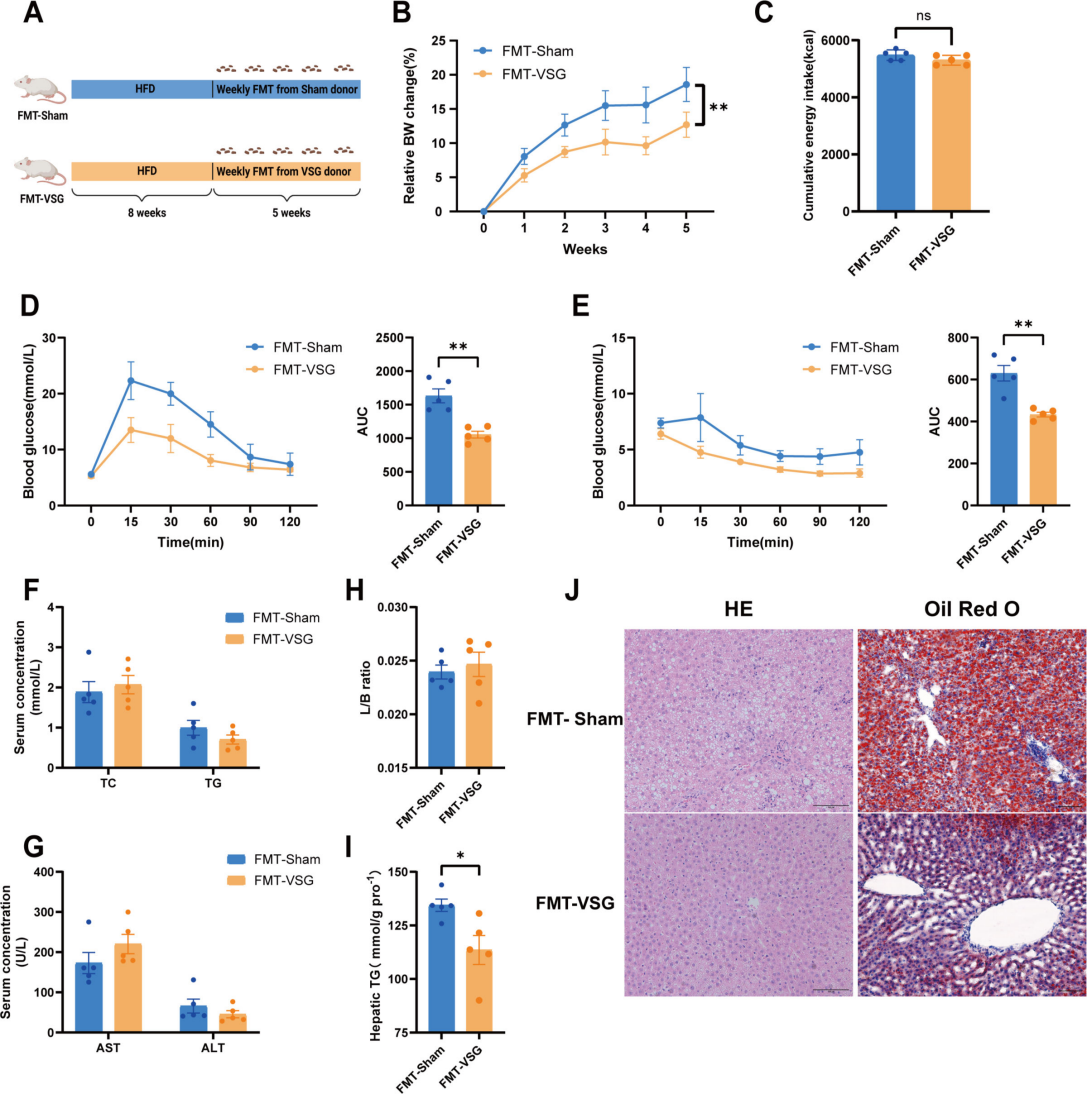
Microbiota transplantation transferred metabolic improvements resulting from VSG surgery in diet‐induced MASLD. (**A**) Illustration of the FMT treatment experiment. Diagram created via BioRender. (**B**) Relative body weight change. (**C**) Cumulative energy intake. (**D**) Glucose tolerance test with the corresponding areas under the curve (AUCs). (**E**) Insulin tolerance test with the corresponding AUCs. (**F**) Serum TC and TG levels. (**G**) Serum AST and ALT levels. (**H**) Liver:body weight ratio. (**I**) Liver TG contents in the FMT-sham and FMT-VSG groups. (**J**) Representative images of H&E staining and Oil Red O staining of rat liver tissues. *n* = 5–6 individuals per group. Each point represents an individual rat. The data are presented as the means ± SDs. *P < 0.05, **P < 0.01, ***P < 0.001, ****P < 0.0001. The data were analyzed via the Mann-Whitney *U*-test.

Second, FMT was performed. The recipient rats were fed an HFD for 8 weeks and then transplanted with either the gut microbiota from the VSG or sham group for an additional 5 weeks (Fig. 4A). We found that weight gain was significantly lower in recipient rats that received the VSG microbiota than in those that received the sham microbiota (Fig. 4B). Food intake did not differ significantly between the FMT-VSG and FMT-sham groups. (Fig. 4C). Transplantation of the VSG microbiota also significantly improved the glucose and insulin tolerance of the recipients (Fig. 4D and E) and reduced hepatic lipid accumulation (Fig. 4I and J) but did not affect the liver-to-body weight ratio or the serum levels of TG, TC, ALT, or AST (Fig. 4F through H). These results show that FMT could mimic the beneficial effects of VSG, indicating that the gut microbiota mediates the metabolic benefits of VSG.

### VSG and FMT reshaped the gut microbiota and enhanced BSH activity in MASLD rats

**Fig 5:**
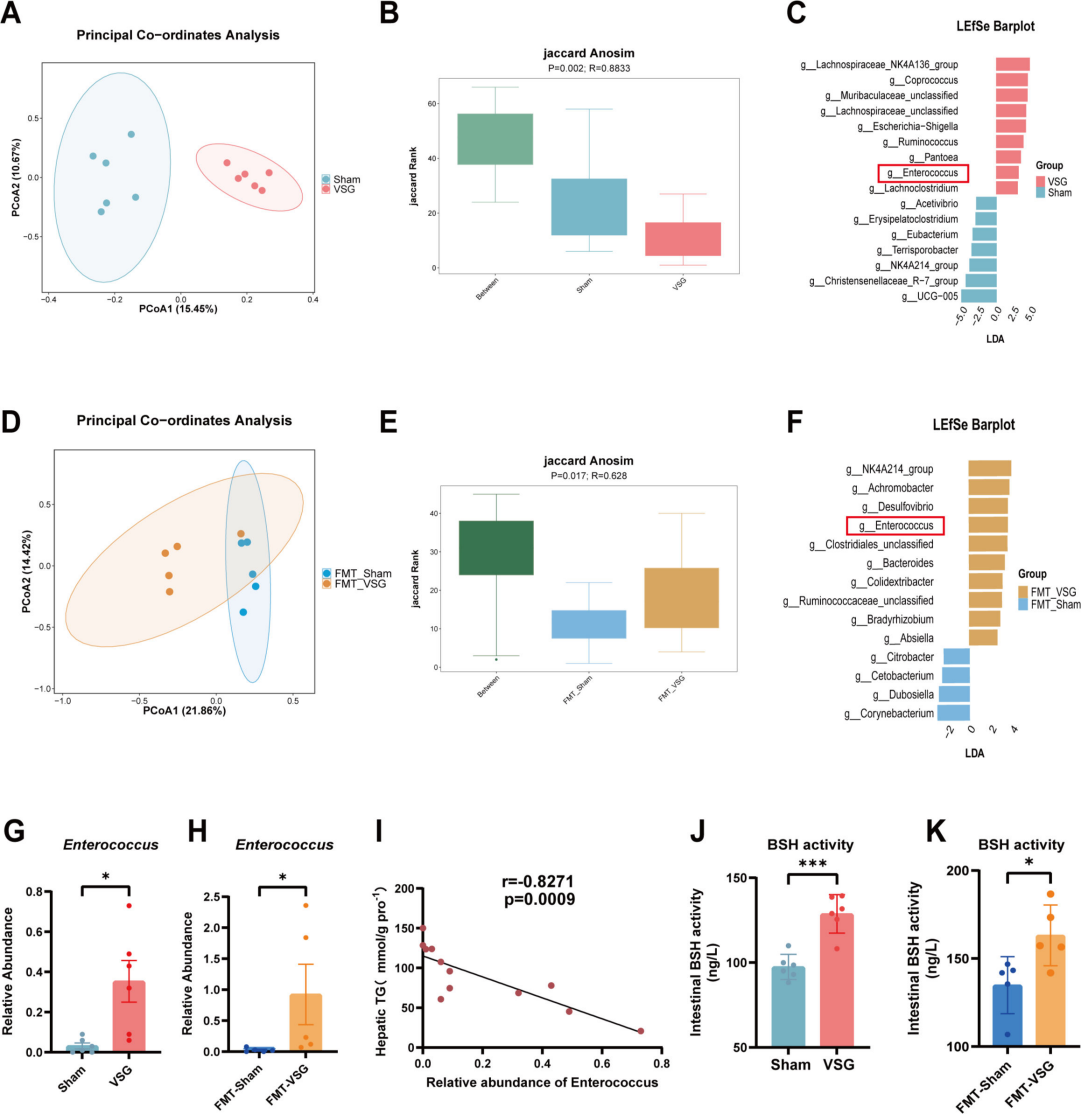
Modulation of the gut microbiome by VSG surgery and VSG microbiota transfer (FMT-VSG) in HFD‐induced MASLD. (**A**) UniFrac distance-based PCoA of microbial communities among the sham and VSG groups. (**B**) Boxplot of the Jaccard rank index Jaccard analysis of similarities (Jaccard ANOSIM) for gut microbiota dissimilarity between the sham and VSG groups. Compared with the sham and VSG groups, the between-group presented significantly greater Jaccard rank values, indicating substantial differences in microbiota composition between the two groups. ANOSIM confirmed significant differences among the groups (*P* = 0.002). (**C**) Comparison of the relative abundance of bacteria at the genus level between the sham and VSG groups was performed via LEfSe. Taxa meeting a linear discriminant analysis (LDA) score threshold >2.5 are shown. (**D**) UniFrac distance-based PCoA of microbial communities among the FMT-Sham and FMT-VSG groups. (**E**) Boxplot of the Jaccard rank index (Jaccard ANOSIM) for gut microbiota dissimilarity between the FMT-sham and FMT-VSG groups. ANOSIM confirmed significant differences among the groups (*P* = 0.017). (**F**) Comparison of the relative abundance of bacteria at the genus level between the FMT-sham and FMT-VSG groups was performed via LEfSe. Taxa meeting an LDA score threshold >2 are shown. (**G**) The relative abundance of *Enterococcus* in the sham and VSG groups. (**H**) The relative abundance of *Enterococcus* in the FMT-sham and FMT-VSG groups. (**I**) Correlation analysis of hepatic TG levels and the relative abundance of *Enterococcus* by Pearson correlation. (**J**) The activity of intestinal BSH in the sham and VSG groups. (**K**) The activity of intestinal BSH in the FMT-sham and FMT-VSG groups. *n* = 5 individuals per group. Each point represents an individual rat. The data are presented as the means ± SDs. *P < 0.05, **P < 0.01, ***P < 0.001, ****P < 0.0001. The data were analyzed via the Mann-Whitney *U*-test.

The alterations in the gut bacterial composition induced by VSG were characterized via 16S rRNA sequencing analysis of the cecal contents. UniFrac distance-based principal coordinate analysis (PCoA) revealed distinct clustering of intestinal microbial communities in the sham and VSG groups (Fig. 5A). The Jaccard rank between the two groups was much greater than that within each group (Fig. 5B). At the genus level, linear discriminant analysis effect size (LEfSe) analysis revealed an obvious alteration in the microbiota, characterized by the enrichment advantages of *Acetivibrio, Erysipelatoclostridium, Eubacterium, Terrisporobacter,* NK4A214_group*, Christensenellaceae*_R-7_Group*,* and UCG_005 in the sham group. *Coprococcus, Muribaculaceae_*unclassified*, Lachnospiraceae_*NK4A136_group*, Lachnospiraceae_*unclassified*, Escherichia-Shigella, Ruminococcus, Pantoea,* and *Enterococcus* were significantly enriched in the VSG group (Fig. 5C).

Furthermore, we compared the cecal microbial composition between the FMT-sham and FMT-VSG groups. Beta diversity analysis, as assessed by PCoA and the Jaccard rank, demonstrated that the transfer of the VSG-altered microbiota significantly modified the microbial signature (Fig. 5D and E). LEfSe analysis revealed obvious microbial alterations at the genus level, characterized by the enrichment of *Corynebacterium, Dubosiella, Cetobacterium,* and *Citrobacter* in the FMT-sham group. NK4A214_group, *Achromobacter*, *Desulfovibrio, Enterococcus*, *Clostridiales_*unclassified*,* and *Bacteroides* were significantly enriched in the FMT-VSG group (Fig. 5F). We found that the genus *Enterococcus* was the only gut microbiota that increased in both the VSG and FMT-VSG groups (Fig. 5G and H), and it was negatively associated with the hepatic TG level (Fig. 5I). *Enterococcus* species are notable for their BSH activity, which contributes to their potential health benefits (10). We found that the BSH activities in the cecal contents were increased significantly in the VSG group and FMT-VSG group (Fig. 5J and K). These findings indicate that VSG can significantly alter the gut microbiota composition in MASLD rats, leading to an increase in BSH activity. Importantly, this effect can be transferred through FMT, suggesting that the metabolic improvements observed following VSG may be mediated by changes in the gut microbiota.

### VSG expanded the pool of unconjugated bile acids and significantly activated the intestinal FXR-FGF19 pathway

In view of the significant changes in the gut microbiota after VSG, especially the BSH-enriched bacteria, we explored the alterations in bile acid (BA) enterohepatic circulation in the sham and VSG groups. We found that bile acid levels significantly increased in the serum, portal blood, and ileum (Fig. S3A, B, and E) and greatly decreased in the cecal contents (Fig. S3D). However, the levels of hepatic bile acids did not differ between the sham and VSG groups (Fig. S3C). To further investigate the synthesis and transport of bile acids, we detected molecules related to them in the liver. We found that the mRNA levels of molecules involved in bile acid synthesis, including CYP7A1, CYP7B1, CYP27A1, BACS, and BAAT, were not significantly different (Fig. S3F). Among the molecules involved in bile acid transport, MRP3 and MRP4, which transport bile acids from the liver to the peripheral blood, and NTCP, which transports bile acids from the portal vein into the liver, significantly increased at the mRNA level in the VSG group (Fig. S3G). In addition, the key molecule for bile acid intestinal reabsorption, ASBT, was significantly increased in the ileum of the VSG group (Fig. S3H through J). These results indicate that VSG affects the enterohepatic circulation of bile acids by promoting their reabsorption in the distal ileum and increasing their transport from the liver to the peripheral blood.

**Fig 6:**
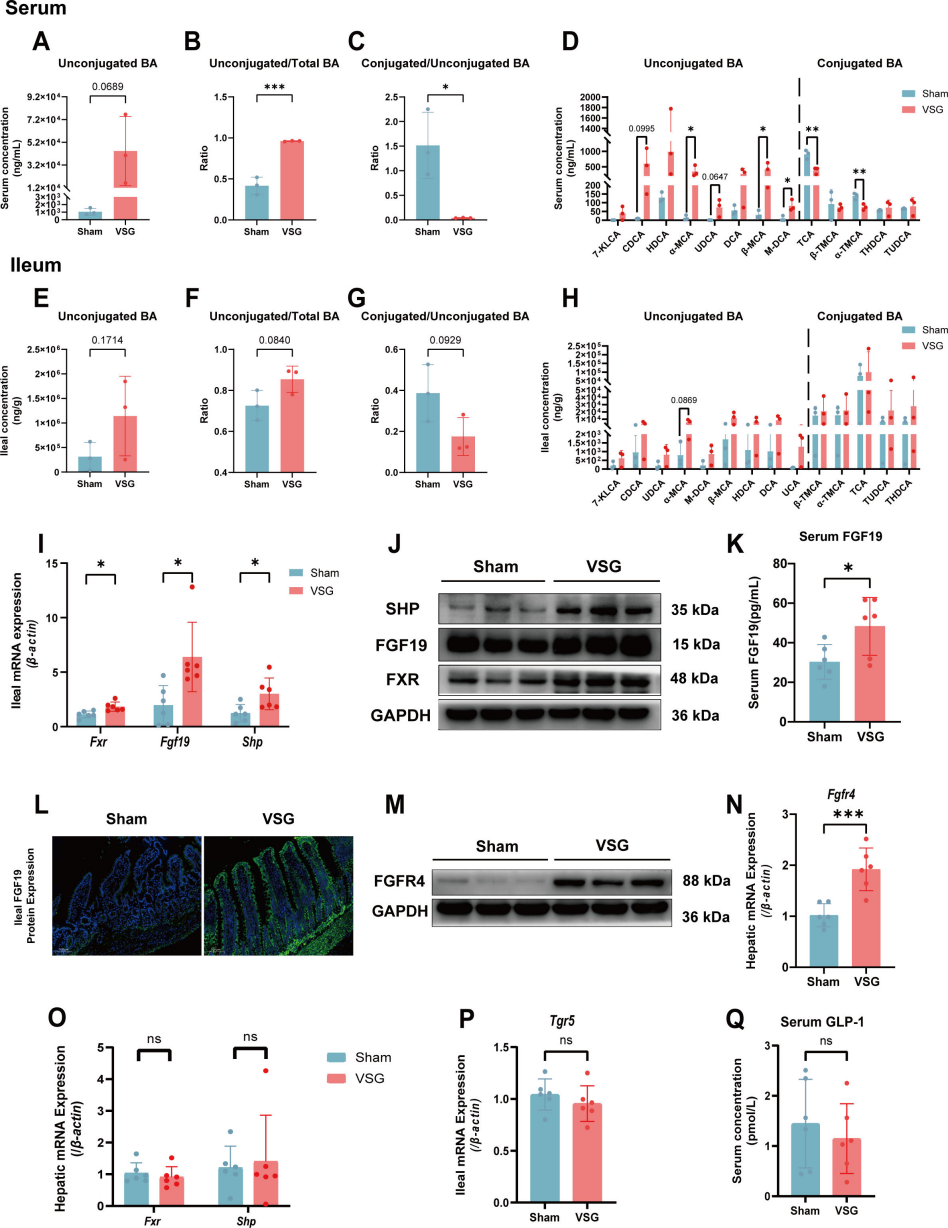
VSG induced the accumulation of unconjugated BAs in the serum and ileum and activated the intestinal FXR-FGF19 pathway. (**A**) Serum unconjugated bile acid levels. (**B**) The ratio of unconjugated to total bile acid. (**C**) The ratio of conjugated to unconjugated bile acid. (**D**) Serum conjugated and unconjugated bile acid levels in the sham and VSG groups. (**E**) Ileal unconjugated bile acid levels. (**F**) The ratio of unconjugated to total bile acid. (**G**) The ratio of conjugated to unconjugated bile acid. (**H**) Ileal conjugated and unconjugated bile acids in the sham and VSG groups. *n* = 3 individuals per group. (**I**) Relative ileal mRNA expression of *Fxr*, *Fgf19*, and *Shp*. (**J**) Relative ileal protein expression of FXR, FGF19, and SHP. (**K**) Serum levels of FGF19 in the sham and VSG groups. (**L**) Immunofluorescence staining of FGF19 in the ileum of the sham and VSG groups. (**M**) Relative hepatic protein expression of FGFR4. (**N**) Relative hepatic mRNA expression of *Fgfr4*. (**O**) Relative hepatic mRNA expression of *Fxr* and *Shp* in the sham and VSG groups. (**P**) Relative ileal mRNA expression of *Tgr5* in the sham and VSG groups. (**Q**) Serum concentration of GLP-1 in the sham and VSG groups. *n* = 6 individuals per group. Each point represents an individual rat. The data are presented as the means ± SDs. *P < 0.05, **P < 0.01, ***P < 0.001, ****P < 0.0001. The data were analyzed via unpaired Student’s *t*-test.

To further elucidate the impact of VSG on bile acid metabolism, which is closely linked to BSH activity, we performed targeted bile acid sequencing of both peripheral blood and ileal tissue samples (11). The levels of unconjugated bile acids in the serum tended to increase in the VSG group (Fig. 6A), and the proportion of unconjugated bile acids in the total bile acids increased markedly in the VSG group (Fig. 6B). Additionally, the ratio of conjugated to unconjugated bile acids in the serum decreased significantly, indicating substantial expansion of the unconjugated bile acid pool in the serum bile acid profile (Fig. 6C). The serum concentrations of unconjugated bile acids, including chenodeoxycholic acid, α-muricholic acid, β-muricholic acid, ursodeoxycholic acid, and murideoxycholic acid, were greater in the VSG group than in the sham group (Fig. 6D). The serum levels of conjugated bile acids, including taurocholic acid and tauro-β-muricholic acid, were significantly decreased in the VSG group (Fig. 6D). In the ileal tissue, there was a trend toward an increase in both the total concentration of unconjugated bile acids and their proportion within the total bile acid pool (Fig. 6E and F). Conversely, the ratio of conjugated to unconjugated bile acids tended to decrease (Fig. 6G). However, the concentration of bile acids in the ileum did not significantly differ (Fig. 6H). The changes in BA composition in the serum and distal ileum further suggest that VSG expanded the pool of unconjugated bile acids in the serum and ileal tissue.

Since FXR is an important bile acid receptor, intestinal FXR can be activated by unconjugated bile acids and further promotes the production of FGF19, which participates in host metabolism (12). We examined the expression of intestinal FXR, SHP, and FGF19 and found that the mRNA and protein levels of intestinal FXR, SHP, and FGF19 were significantly increased in the VSG group (Fig. 6I and J), and these findings were confirmed by immunofluorescence (Fig. 6L). However, the expression levels of hepatic FXR and SHP did not differ significantly (Fig. 6O). After FGF19 is released into the portal vein, it further activates the hepatic FGFR4 receptor and participates in metabolic regulation. We found that hepatic FGFR4 mRNA and protein expression increased in parallel with the increase in the serum FGF19 concentration, as measured by enzyme-linked immunosorbent assay (ELISA), in the VSG group (Fig. 6K, M, and N). We also measured the levels of another intestinal bile acid receptor, TGR5, and peripheral blood GLP-1 and found no significant differences (Fig. 6P and Q). These results indicate that VSG induced the accumulation of unconjugated BAs, which in turn significantly activated the intestinal FXR-FGF19 pathway.

### VSG microbiota transfer increased unconjugated bile acid levels and modulated intestinal FXR-FGF19 signaling in HFD-induced MASLD

**Fig 7:**
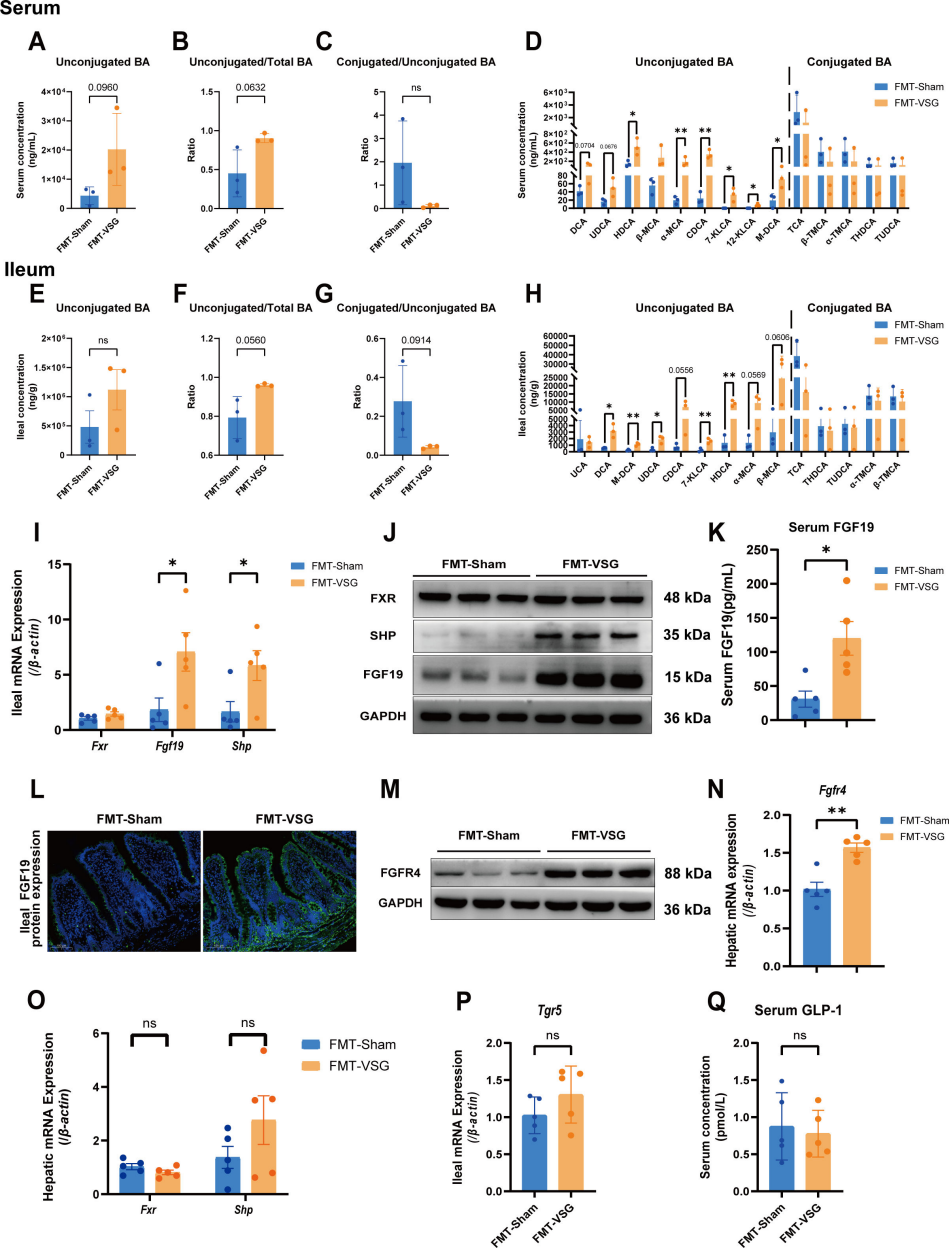
Microbiota transplantation of VSG induced the accumulation of unconjugated BAs in the serum and ileum and activated the intestinal FXR-FGF19 pathway. (**A**) Serum unconjugated bile acid levels. (**B**) The ratio of unconjugated to total bile acid. (**C**) The ratio of conjugated to unconjugated bile acid. (**D**) Serum conjugated and unconjugated bile acids in the FMT-sham and FMT-VSG groups. (**E**) Ileal unconjugated bile acid levels. (**F**) The ratio of unconjugated to total bile acid. (**G**) The ratio of conjugated to unconjugated bile acid. (**H**) Ileal conjugated and unconjugated bile acids in the FMT-sham and FMT-VSG groups. *n* = 3 individuals per group. (**I**) Relative ileal mRNA expression of *Fxr*, *Fgf19*, and *Shp*. (**J**) Relative ileal protein expression of FXR, FGF19, and SHP. (**K**) Serum levels of FGF19 in the FMT-sham and FMT-VSG groups. (**L**) Immunofluorescence staining of FGF19 in the ileum of the FMT-sham and FMT-VSG groups. (**M**) Relative hepatic protein expression of FGFR4. (**N**) Relative hepatic mRNA expression of *Fgfr4*. (**O**) Relative hepatic mRNA expression of *Fxr* and *Shp* in the FMT-sham and FMT-VSG groups. (**P**) Relative ileal mRNA expression of *Tgr5* in the FMT-sham and FMT-VSG groups. (**Q**) Serum concentrations of GLP-1 in the FMT-sham and FMT-VSG groups. *n* = 5 individuals per group. Each point represents an individual rat. The data are presented as the means ± SDs. *P < 0.05, **P < 0.01, ***P < 0.001, ****P < 0.0001. The data were analyzed via the Mann-Whitney *U*-test.

Having established a causal relationship between the VSG-associated microbiota and host metabolic balance, we aimed to further elucidate the mechanisms underlying the interaction between the gut microbiota and the host that collectively contribute to weight reduction and metabolic reconfiguration in MASLD host organisms. We examined the changes in the composition of bile acids in the serum and distal ileum, as well as the impact on bile acid receptors, following the transfer of the gut microbiota from VSG donors (11). We observed that the levels of unconjugated bile acids in the serum and distal ileal tissue did not change significantly (Fig. 7A and E), but the proportion of unconjugated bile acids in total bile acids increased in the FMT-VSG group (Fig. 7B and F). However, the ratio of conjugated to unconjugated bile acids in the serum and ileal tissues did not differ (Fig. 7C and G). In the FMT-VSG group, the serum levels of unconjugated bile acids, including hyodeoxycholic acid, chenodeoxycholic acid, α-muricholic acid, β-muricholic acid, 7-ketolithocholic acid, 12-ketolithocholic acid, and homicoxycholic acid, were significantly increased (Fig. 7D). In contrast, the conjugated bile acids were not significantly different (Fig. 7D). In ileal tissue, the levels of unconjugated bile acids, specifically deoxycholic acid, ursodeoxycholic acid, chenodeoxycholic acid, 7-ketolithocholic acid, hyodeoxycholic acid, and murideoxycholic acid, also significantly increased, whereas the level of conjugated bile acids did not significantly change (Fig. 7H). The transfer of the gut microbiota from VSG rats resulted in an increase in unconjugated bile acids similar to those induced by VSG, further suggesting a causal role of the gut microbiota in the changes in the bile acid profile caused by VSG.

A significant increase in unconjugated bile acids, particularly chenodeoxycholic acid (CDCA), can markedly activate the bile acid receptor FXR and its downstream signaling molecules. Therefore, we investigated the FXR pathway in the FMT group. Hepatic FXR and SHP expression levels did not differ (Fig. 7O). The results of qRT-PCR and western blot (WB) confirmed that the intestinal FXR-FGF19 pathway was significantly activated in the FMT-VSG group, together with an increased serum concentration of FGF19 (Fig. 7I through K). The immunofluorescence results also revealed an increase in the ileal FGF19 level (Fig. 7L). Moreover, the mRNA and protein levels of FGFR4 in the liver increased significantly in the FMT-VSG group (Fig. 7M and N). Additionally, there were no significant differences in the levels of the TGR5 pathway and its induced GLP-1 between the FMT-sham and FMT-VSG groups (Fig. 7P and Q). These findings demonstrate that transplantation of the VSG-associated microbiome, characterized by increased BSH activity, significantly elevated the levels of unconjugated bile acids. The increased pool of unconjugated bile acids, particularly those acting as potent agonists of FXR, robustly activates the intestinal FXR-FGF19 signaling pathway, thereby driving the observed metabolic benefits.

### The VSG gut microbiota requires intestinal FXR signaling to exert metabolic benefits in HFD-induced MASLD

**Fig 8:**
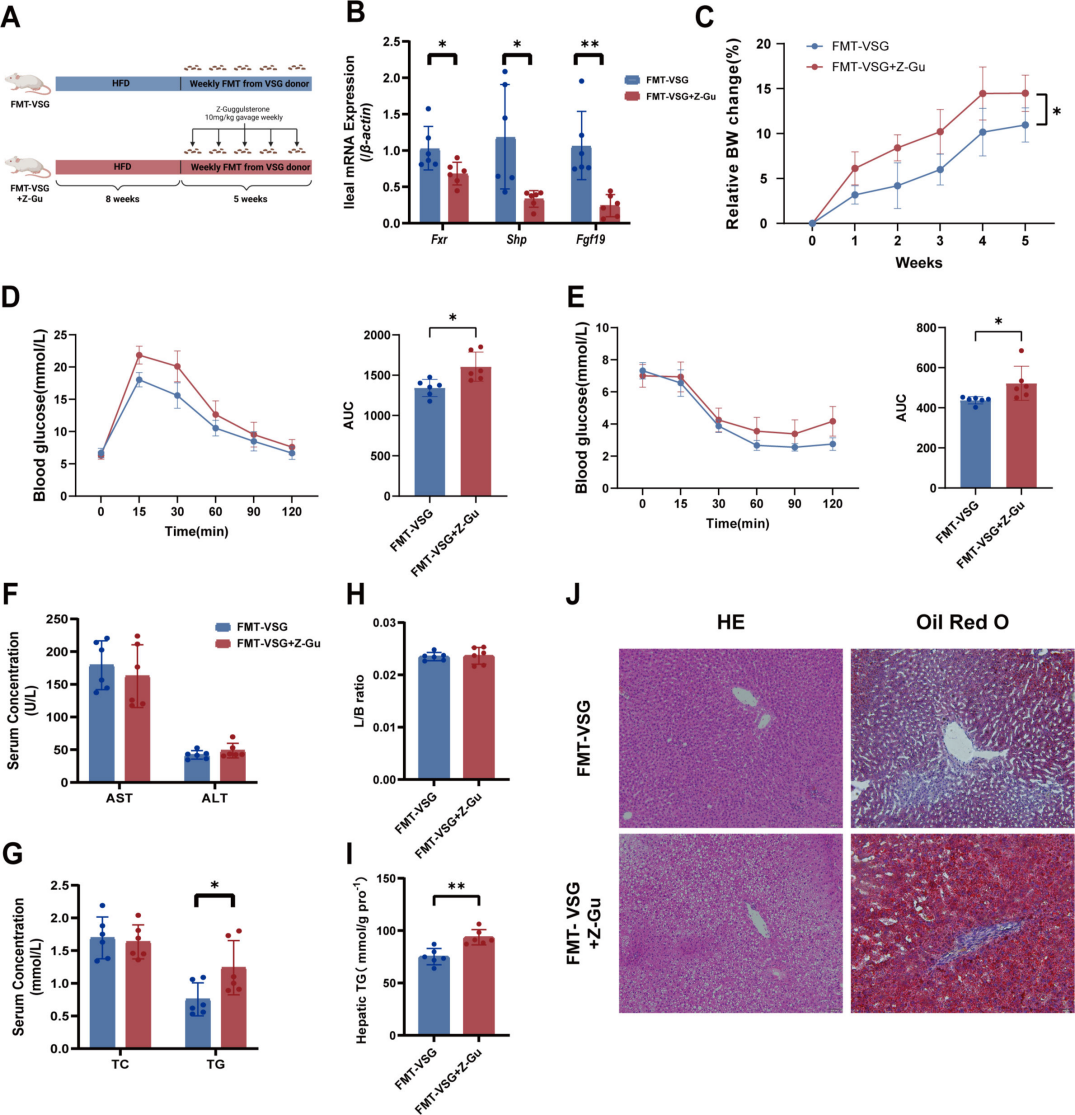
The VSG microbiota requires intestinal FXR to transfer metabolic health benefits in HFD‐induced MASLD. (**A**) Illustration of the FXR inhibition treatment experiment. Diagram created via BioRender. (**B**) Relative ileal mRNA expression of *Fxr, Fgf19,* and *Shp.* (**C**) Relative body weight change. (**D**) Glucose tolerance test with the corresponding areas under the curve (AUCs). (**E**) Insulin tolerance test with the corresponding AUCs. (**F**) Serum AST and ALT levels. (**G**) Serum TG and TC levels. (**H**) Liver:body weight ratio and (**I**) liver TG contents. (**J**) Representative images of H&E staining and Oil Red O staining of rat liver tissues. *n* = 6 individuals per group. Each point represents an individual rat. The data are presented as the means ± SDs. *P < 0.05, **P < 0.01, ***P < 0.001, ****P < 0.0001. The data were analyzed via unpaired Student’s *t*-test.

To confirm the role of intestinal FXR in the metabolic benefits of the VSG microbiome, a follow-up recovery study in which HFD-fed rats were subjected to transplantation of the VSG microbiota and VSG microbiota coupled with Z-guggulsterone, an FXR antagonist, for 5 weeks was performed (Fig. 8A)(13). Suppression of intestinal FXR signaling by Z-guggulsterone (Fig. 8B) reduced the beneficial effects of the VSG microbiota on relative body weight changes (Fig. 8C) and glucose homeostasis (Fig. 8D and E) in the FMT-VSG+Z-Gu group. Furthermore, suppression of intestinal FXR signaling significantly reduced the beneficial effects of VSG microbiota transfer on serum TG levels and hepatic steatosis (Fig. 8G, I, and J) but did not affect the liver-to-body weight ratio or the serum levels of TC, ALT, or AST (Fig. 8F through H). These findings indicate that intestinal FXR signaling mediates the metabolic benefits of VSG microbiota transplantation in MASLD rats.

## DISCUSSION

This study demonstrated that VSG significantly improved the gut microbiota, especially by increasing BSH activity, in MASLD rats. Increased BSH activity significantly increased the proportion of FXR-agonistic bile acids and further activated the intestinal FXR-FGF19 axis, thereby improving MASLD. These findings explored the key roles and mechanisms of the gut microbiota in the metabolic benefits of VSG.

As the most common type of bariatric surgery, VSG can result in long-term weight loss and metabolic improvement, independent of body weight (14). We found that the metabolic benefits of VSG on MASLD extend beyond calorie restriction, implicating the involvement of additional mechanisms, including increased energy expenditure and attenuated lipid absorption (15–17). Studies have shown that the gut microbiota changes significantly after VSG and is strongly associated with metabolic benefits (18). However, most studies have focused only on changes in the gut microbiota after VSG without further exploration of the causal relationship between the gut microbiota and the metabolic benefits of VSG. In this study, we emphasized two distinct implications of the gut microbiota for the metabolic benefits of VSG. First, by eliminating the gut microbiota after VSG, we found that the effects of VSG on glucose homeostasis and liver steatosis disappeared, indicating that the gut microbiota is necessary for the metabolic benefits of VSG. Additionally, our antibiotic intervention experiments in SCD-fed rats ruled out potential direct metabolic effects of antibiotics themselves, thereby providing evidence that the elimination of the gut microbiota partially mediates the metabolic benefits observed after VSG surgery. Second, by transferring the gut microbiota after VSG through fecal microbiota transplantation, we found that gut microbiota transplantation produced an anti-MASLD phenotype similar to that of VSG in MASLD rats. These findings indirectly indicate that microbiota transplantation from VSG donors may be a promising new therapy for MASLD treatment.

The mechanism by which the gut microbiota acts in VSG is unclear. The BSH enzyme is involved in the “gateway” reaction of conjugated bile acids during bacterial metabolism. Its main function is to catalyze the deconjugation of conjugated bile acids to release free primary bile acids, which is a prerequisite for the subsequent production of secondary BAs via 7α/β-dehydroxylation reactions (19). BSH can reduce the intestinal digestion rate of fats, thereby reducing energy absorption and weight gain (20). Studies have shown that gut bacterial strains with high BSH expression, such as *Lactobacillus plantarum* H-87 and *Lactobacillus reuteri* J1, have significant antiobesity effects. They can improve bile acid homeostasis and ameliorate obesity by regulating bile acid receptor pathways (21). A previous study revealed that the abundance of BSH-enriched *Lactobacillus* was significantly increased in VSG mice (22). However, the relationship between BSH activity and the metabolic benefits of VSG remains unclear. Our study revealed that after VSG, the abundance of *Enterococcus*, which produces BSH, significantly increased and was negatively correlated with the accumulation of liver TG. Additionally, increased BSH activity is involved in bile acid metabolism, significantly expanding the pool of unconjugated bile acids in the serum and distal ileum. A similar phenomenon was observed in MASLD rats that received gut microbiota transfer from the VSG, indicating that increased BSH activity is a potential target for the involvement of the gut microbiota in metabolic improvements in the VSG.

The enterohepatic circulation of bile acids is involved in maintaining systemic metabolic homeostasis, with the gut microbiota playing a crucial role. Bile acids, as endogenous signaling molecules, can bind to bile acid receptors, such as FXR and TGR5, to regulate bile acid homeostasis, participate in lipid metabolism, and maintain energy homeostasis (20). Lithocholic acid (LCA) has the strongest effect on TGR5 activation, while CDCA has the highest capacity to activate FXR (23). TGR5, a member of the rhodopsin-like subfamily of G-protein coupled receptors (GPCRs), participates in controlling lipid metabolism, glucose homeostasis, and energy expenditure (24). Specifically, in intestinal enteroendocrine L cells, TGR5 activation stimulates the secretion of GLP-1, an incretin hormone that ameliorates systemic glucose metabolism and hepatic lipid accumulation in obese mice (25). Notably, both VSG surgery and VSG-derived fecal microbiota transplantation resulted in significantly elevated CDCA concentrations in the ileal tissue and serum, whereas the LCA levels remained unchanged. Additionally, ileal TGR5 expression and systemic GLP-1 levels did not significantly change following either intervention. Therefore, we further examined the FXR pathway in MASLD rats that underwent VSG surgery or received gut microbiota transplantation from the VSG. We found that the expression of intestinal FXR increased significantly and that its downstream molecules, FGF19 and SHP, were also markedly activated. As a member of the fibroblast growth factor family, FGF19 is expressed primarily in the small intestine. Upon activation of FXR, FGF19 is released into the portal vein and exerts its effects in the liver by binding to the FGFR4 receptor (26). FGF19 has been shown to be involved in improving systemic glucose and lipid metabolism and alleviating MASLD through various pathways (27). The overexpression of FGF19 can improve the accumulation of liver lipids and alleviate fatty liver by attenuating the expression of sterol regulatory element-binding protein 1c and ACC in the liver (28). Exogenous supplementation with recombinant FGF19 protected *ob/ob* mice from diabetes and improved glucose metabolism (29). Studies have indicated that circulating FGF19 levels are significantly reduced in obese patients and markedly increase after bariatric surgery, suggesting that FGF19 may be a target through which weight loss surgery improves obesity (30). Our results suggest that the VSG microbiome, with its increased BSH activity, expands the pool of unconjugated bile acids, which activate the intestinal FXR-FGF19 pathway and mediate metabolic benefits. To further clarify the role of the intestinal FXR-FGF19 signaling pathway after VSG surgery, we inhibited intestinal FXR while performing gut microbiota transplantation from the VSG. Pharmacological inhibition of intestinal FXR by Z-guggulsterone can ameliorate HFD-induced MASLD phenotypes, mirroring the metabolic improvements observed in intestine-specific FXR knockout mice (13). The results showed that the suppression of intestinal FXR eliminated the beneficial effects of the VSG gut microbiota on hepatic steatosis and glucose homeostasis, highlighting that the intestinal FXR-FGF19 pathway may play an important role in the metabolic improvements associated with the transfer of the VSG microbiota.

The optimal therapeutic strategy for targeting FXR in MASLD remains controversial. Emerging evidence suggests that intestine-specific FXR agonists may ameliorate hepatic steatosis through BA modulation, the suppression of hepatic gluconeogenesis, and the promotion of white adipose tissue thermogenesis, independent of hepatic FXR activation (31). In contrast, pharmacological inhibition of the intestinal FXR-ceramide signaling pathway has also demonstrated significant efficacy in attenuating liver fat accumulation (13). This contradictory phenomenon illustrates the complex role of intestinal FXR as a sensor of the gut microbiota in regulating systemic metabolism, and the activation and inhibition of intestinal FXR may lead to similar metabolic improvements within the body (32).

This study had several limitations. First, owing to the varying colonization abilities of different bacteria in donor feces, the microbial composition of the recipient rats was not exactly the same as that of the donor rats. In future experiments, germ-free mice should be used. Second, shorter weight monitoring intervals would better delineate the dynamic patterns of the VSG and pair-feeding effects on body weight. Third, while existing studies have shown that both global FXR knockout and intestinal FGF15 deletion abolish the metabolic benefits of VSG, the tissue-specific effects of FXR on the VSG-remodeled gut microbiota remain unknown (33, 34); therefore, intestinal-specific FXR knockout mice will be employed in our future studies. Additionally, intervention with an FXR agonist in MASLD rats receiving sham-FMT provides complementary evidence that FXR serves as the downstream target through which the VSG-remodeled gut microbiota exerts metabolic benefits, which warrants implementation in future studies. Finally, considering that rats do not have a gallbladder and that their bile acid structure differs from that of humans, further studies are needed to clarify whether the changes in bile acid structure in VSG rats are applicable to humans.

In summary, we demonstrated that VSG increased BSH-enriched gut bacteria and altered bile acid profiles in the distal ileum, which further activated the intestinal FXR-FGF19 signaling pathway and improved systemic metabolism. Our study provides new insights into the mechanisms by which the gut microbiota affects systemic metabolic homeostasis restored by VSG surgery and further strengthens the rationale for microbiota-targeted strategies to achieve long-term weight reduction and MASLD reduction.

## MATERIALS AND METHODS

### Animal experiments

Age-matched (5-week-old) male Sprague-Dawley rats (140 g–160 g) were purchased from the Zhejiang Academy of Medical Sciences (Hangzhou, China) and housed at the Animal Center of the First Affiliated Hospital, Zhejiang University School of Medicine. The rats were housed in a specific pathogen-free environment with a 12 h light/dark cycle at a temperature of 21°C–23°C and humidity of 40%–60%.

In the VSG intervention study, the rats were acclimated by providing them with unrestricted access to a standard diet for 1 week and then fed a HFD (60 kcal% fat, D12492, Research Diets Inc., New Brunswick, NJ, USA). After 8 weeks of HFD feeding, the rats were randomly divided into the VSG and sham groups, which received VSG surgery or sham surgery, respectively.

In the pair-fed intervention study, following a 1 week acclimatization period, the rats were fed a HFD for 8 weeks. The rats were randomly assigned to three groups: the sham, VSG, and pair-fed groups. The sham group underwent sham surgery, whereas the pair-fed group received sham surgery and was pair-fed to match the food intake of the VSG group. Following a 1-week postoperative recovery period, the animals were gradually transitioned to an HFD. After this transition, the daily food consumption in the VSG group was meticulously recorded. To ensure precise caloric matching, the pair-fed group was provided a daily food ration equivalent to the amount consumed by the VSG group on the preceding day.

In the oral broad-spectrum antibiotic cocktail (ABX) intervention study, following a 1 week acclimatization period, the rats were fed an HFD for 8 weeks. The rats were then randomly divided into three groups: the sham group, which received sham surgery; the VSG group, which underwent VSG surgery; and the VSG + ABX group, which received ABX after the VSG operation.

To study the effects of antibiotics (ABXs) on systemic metabolism, after 1 week of acclimatization, the rats were randomly divided into two groups: the SCD group, which was fed a standard chow diet (SCD) without ABX, and the SCD + ABX group, which was fed SCD with ABX for 5 weeks.

In the FMT study, the donor rats underwent either sham surgery or VSG. After 1 week of acclimatization, the rats were fed an HFD for 8 weeks. The rats were randomly assigned to two groups: one group (FMT-sham) received fecal supernatant from sham-operated rats weekly, and the other group (FMT-VSG) received fecal supernatant from VSG-operated rats. These rats were fed an HFD for an additional 5 weeks.

For the FXR regulation study, following a 1 week acclimatization period, the rats were fed an HFD for 8 weeks. Before being randomly divided into two groups, the FMT-VSG group received fecal supernatant from the VSG group weekly, and the FMT-VSG + Z-Gu group received fecal supernatant from the VSG group and 10 mg/kg (Z)-guggulsterone by weekly gavage.

All the rats had *ad libitum* access to food and water, and their body weights and food consumption were monitored weekly. Following the experiments, the rats were fasted overnight before euthanasia. Blood samples were collected and centrifuged at 3,000 rpm at 4°C for 10 min to obtain serum. Tissues, including the liver, cecal content, and ileal tissue, were removed from the experiments.

### Statistical analysis

GraphPad Prism version 10.1.1 (GraphPad Software, La Jolla, CA, USA) was used to handle the data acquired from the experiments. The data are presented as the means ± SDs of biological replicates. The normality of each parameter was tested via the Shapiro-Wilk test. Parametric data were compared via unpaired Student’s *t*-test between two groups or one-way analysis of variance with Tukey’s *post hoc* comparisons among multiple groups. The Mann-Whitney *U*-test was used to analyze nonparametric data. Correlations were analyzed via Pearson correlation. A two-tailed *P*-value <0.05 was considered statistically significant.

Additional details are provided in the supplemental material.

## Data Availability

The 16S rRNA amplicon sequencing data were deposited at the China National Genomics Data Center with BioProject IDs PRJCA029587 and PRJCA036121.

## AFTER EPUB

*[This article was published on 20 October 2025 with an error in Shixian Lu's affiliations. The affiliations were corrected in the current version, posted on 28 October 2025.]*

## SUPPLEMENTAL MATERIAL

Supplemental material msystems.00634-25-s0001.docx Additional experimental details and supplemental figures and table.

ASM does not own the copyrights to Supplemental Material that may be linked to, or accessed through, an article. The authors have granted ASM a non-exclusive, world-wide license to publish the Supplemental Material files. Please contact the corresponding author directly for reuse.
