# Supplementary material for: Microbial changes resulting from VSG attenuate MASLD by modulating bile acid metabolism and the intestinal FXR-FGF19 axis
Source: mSystems. 2025 Oct 20;10(11):e00634-25. doi: 10.1128/msystems.00634-25 (PMC12625774; doi:10.1128/msystems.00634-25)
Supplement: Supplemental material — Additional experimental details and supplemental figures and table. [file msystems.00634-25-s0001.docx]

**Supplementary materials**

**Microbial changes resulting from VSG attenuate MASLD by modulating bile acid metabolism and the intestinal FXR‒FGF19 axis**

Yi Xia^a^, Jinpu Yang^a,b^, Shixian Lu^c^, Weixin Cheng^a^, Mengting Ren^a,d^, Zhening Liu^a^, Ling Yang^a^, Qien Shen^a^, Yujie Liang^a^, Hangkai Huang^a^, Minjie Chen^a^, Xinxin Zhou^a^, Mosang Yu^a^, Feng Ji^a,*^#, Chengfu Xu^a,*^#

^a^ Department of Gastroenterology, The First Affiliated Hospital, Zhejiang University School of Medicine, Hangzhou, China

^b^ Department of Gastroenterology, Affiliated Hangzhou First People's Hospital, School of Medicine, Westlake University, Hangzhou, China

^c^ Department of Gastroenterology, Shengzhou Hospital of Traditional Chinese Medicine, Shaoxing, China

^d^ Cancer Center, Department of Gastroenterology, Zhejiang Provincial People's Hospital (Affiliated People's Hospital), Hangzhou Medical College, Hangzhou, China

***Corresponding authors:**

Prof. Feng Ji, Email: [jifeng@zju.edu.cn](mailto:jifeng@zju.edu.cn);

Prof. Chengfu Xu, Email: [xiaofu@zju.edu.cn](mailto:xiaofu@zju.edu.cn)

**Supplementary Methods**

**Antibiotic treatment**

An antibiotic cocktail (ABX, neomycin 1 g/L, metronidazole 1 g/L, vancomycin 0.5 g/L, and 1 g/L ampicillin) was dissolved and freshly supplied to the drinking water for 6 weeks. All antibiotics were purchased from MedChemExpress.

**Fecal microbiota transplantation**

Fresh fecal samples from body weight-stabilized rats (from week 2 postoperatively) were collected in sterile microtubes and stored on ice. The collected feces were homogenized by vortexing in sterile phosphate-buffered saline (PBS) at 100 mg/mL for 2 min, homogenized carefully, and administered via oral gavage with a suspension of 10 ml/kg body weight. The supernatant was prepared 10 min prior to gavage on the day of transplantation to reduce the impact of external factors on the viability of the taxa. The donor rats were the entire group of rats that underwent VSG or sham surgery, with each group originating from the same cage to eliminate the cage effect.

**VSG surgery**

After 12 h of fasting, the rats were anesthetized with 2% isoflurane (RWD; Shenzhen, China), and surgical operations were performed according to standard procedures.

VSG was performed as follows. Initially, a midline incision was made in the upper abdomen to expose the entire stomach and the lower esophagus. This was followed by securing and severing the gastrosplenic ligament and associated vessels. The major branches of the gastric artery and vein were tied with 7–0 monofilament absorbable sutures. Typically, four major vessels per gastric wall require ligation, although anatomical variations are observed between the rats. The gastric transection line was initiated approximately 2 mm above the cardiac notch (toward the greater curvature) and extended at least 2 mm below the margo plicatus, continuing to the proximal end of the right pancreatic lobe. Approximately 80% of the stomach was excised, leaving a tubular segment. The gastric walls were then approximated with 5–0 monofilament, absorbable sutures via a taper needle in a simple interrupted pattern. Finally, the abdominal incision was closed via 3–0 monofilament absorbable sutures.

For the sham procedure, the incision and transection sites were similar to those in the VSG group and were anastomosed in situ. The operative time was the same as that in the VSG group.

After the operation, the rats were given water 24 h later, followed by a fluid diet (Ensure, Shanghai, China) for the next 7 days, after which they were gradually transitioned to a HFD for 1 week.

**Total bile acid assay**

The total bile acid content in the cecal contents, ileal tissue, and liver tissue was determined via a total bile acid detection assay kit (Nanjing Jiancheng Bioengineering Institute).

**ELISA**

FGF19 levels in rat serum were measured via an FGF19 ELISA kit (SEC917Ra, Cloud Clone Corp.), following the manufacturer’s protocol. A rat BSH ELISA kit (MEIMIAN, Wuhan, China) was used to measure the activity of BSH in the cecal contents. Serum levels of glucagon-like peptide-1 (GLP-1) were measured via a glucagon-like peptide-1 (GLP-1) ELISA kit (MEIMIAN, Wuhan, China) following the manufacturer’s protocol.

**Glucose and insulin tolerance tests**

Before the glucose tolerance test (GTT), all the rats were fasted for 16 h and then received an intraperitoneal injection of glucose (1 g/kg body weight; Sigma, St. Louis, MO, USA). For the insulin tolerance test (ITT), the animals were fasted for 6 h prior to intraperitoneal injection of human insulin (1 U/kg body weight; Wanbang, Xuzhou, China). Blood glucose levels were measured at 0, 15, 30, 60, 90, and 120 min post-injection.

**Hematoxylin‒eosin (H&E) and Oil Red O staining and histological analysis**

After the rats were euthanized, their tissues were immediately removed and immersed in cold PBS. The liver and intestine were preserved in 4% paraformaldehyde for 24 h and embedded in paraffin. The tissue sections were subjected to hematoxylin and eosin (H&E) and immunofluorescence staining.

For immunofluorescence staining, an anti-FGF19 antibody (sc-398338; Santa Cruz Biotechnology) was used.

For Oil Red O staining, the tissues were embedded in optimal cutting temperature (OCT) compound and quickly frozen. The sections were warmed, treated with 0.3% Oil Red O solution, and thoroughly washed with water. The stained sections were visualized under an inverted microscope (Olympus, Tokyo, Japan).

**Serum biochemical indexes**

Aspartate aminotransferase (AST), alanine transaminase (ALT), total triglyceride (TG), total cholesterol (TC), and total bile acid (BA) levels in the serum were detected via an automatic biochemical analyzer (HITACHI, Tokyo, Japan).

**Hepatic triglyceride assay**

The intrahepatic triglyceride content was determined via a commercial kit (Applygen Technologies Inc., Beijing, China) according to the manufacturer's instructions. Briefly, the liver sections were homogenized in lysis buffer. Suitable amounts of homogenates were transferred for TG analysis. Triglyceride levels were normalized to the protein content in the same tissue.

**16S sequencing analysis**

16S rRNA sequencing analysis was performed as previously described(1). Total RNA was isolated from the cecal contents (50 mg) via a QIAamp DNA Stool Mini Kit (QIAGEN) following the manufacturer’s protocol. The V3-V4 hypervariable region of the bacterial 16S rRNA gene was amplified with the primers 341 F (5’-CCTACGGGNGGCWGCAG-3’) and 805 R (5’-GACTACHVGGGTATTCTAATCC-3’). The PCR amplification products were evaluated via 2% agarose gel electrophoresis, and the target gene fragments were recovered via the AxyPrep PCR Clean-up Kit (Axygen) following the manufacturer’s instructions. The Quant-iT PicoGreen dsDNA Assay Kit (Invitrogen) was used to quantify the libraries of the purified PCR products via a Qubit Fluorometric Quantitation System. The sequencing primers were removed from the demultiplexed raw sequences via Cutadapt (v1.9). The paired-end reads were subsequently merged via FLASH (v1.2.8). Low-quality reads (quality scores<20), short reads (<100 bp), and reads containing more than 5% “N” records were trimmed via the sliding-window algorithm in fqtrim (v 0.94). Quality filtering was performed to obtain high-quality clean tags according to fqtrim. Chimeric sequences were filtered via Vsearch software (v2.3.4). DADA2 was used to denoise and generate amplicon sequence variants (ASVs). Sequence alignment of species annotations was performed via the QIIME2 plugin feature classifier, and the alignment databases used were SILVA and NT-16S. Alpha and beta diversities were also calculated via QIIME2. PERMANOVA (1,000 Monte Carlo permutations) based on Bray‒Curtis and Jaccard dissimilarities was performed to determine the microbial differences between groups, and the relative abundance was used in bacterial taxonomy. The Wilcoxon test was used to identify differentially abundant genera, and significant differences were considered at *P*<0.05. Linear discriminant analysis (LDA) effect size (LEfSe, LDA ≥ 2.5, P < 0.05) was performed via LEFSe software. Other diagrams were implemented via the R package (v3.4.4).

**Targeted metabolomic analysis of bile acid**

The BAs in the ileal tissue and serum were analyzed as previously described. Briefly, weighed ileal tissue was resuspended, sonicated, and homogenized in cold extraction solutions with methanol and acetonitrile. Serum was obtained by centrifugation (3000 rpm for 10 min). The supernatant was then filtered through a 0.22 μm sterile membrane and examined via a UPLC-ESI-MS/MS system (Waters, Milford, MA) according to standard protocols. D4 Cholic acid was used as the internal standard.

**Western blot analysis**

Total protein was extracted via RIPA buffer (FD008) containing protease and phosphatase inhibitors (FUDE). The extracted proteins were quantified via a BCA kit (P0011, Beyotime). Equal amounts of protein were separated by 10% SDS–PAGE, followed by transfer to PVDF membranes (Millipore, Inc., Darmstadt, Germany). After blocking with 5% nonfat milk, the membrane was incubated at 4°C with the following primary antibodies: anti-FGF19 (sc-398338) and anti-FXR (sc-25309) antibodies purchased from Santa Cruz. Anti-SHP (A16454) was purchased from ABclonal. Anti-GAPDH (60004-1-Ig) and anti-FGFR4 (11098-1-AP) antibodies were purchased from Proteintech. After being rinsed with TBST three times, the slides were further incubated with HRP-conjugated secondary antibodies (Biosharp). Proteins were visualized via an enhanced chemiluminescence (ECL) Plus kit (FD8020, FUDE).

**Quantitative real-time PCR**

The RNA was prepared via the use of TRIzol Reagent and then reverse transcribed into cDNA. Quantitative real-time polymerase chain reaction (qPCR) was performed via SYBR Green Pro Taq (Accurate Biotechnology [Hunan] Co., Ltd., Hunan, China). Relative RNA levels were normalized to those of β-actin. The primer sequences are listed in Table S1.

**References**

1. Li Y, Wang Y, Shi F, Zhang X, Zhang Y, Bi K, Chen X, Li L, Diao H. 2022. Phospholipid metabolites of the gut microbiota promote hypoxia-induced intestinal injury via CD1d-dependent γδ T cells. Gut Microbes 14:2096994.

**Supplementary figures**

**
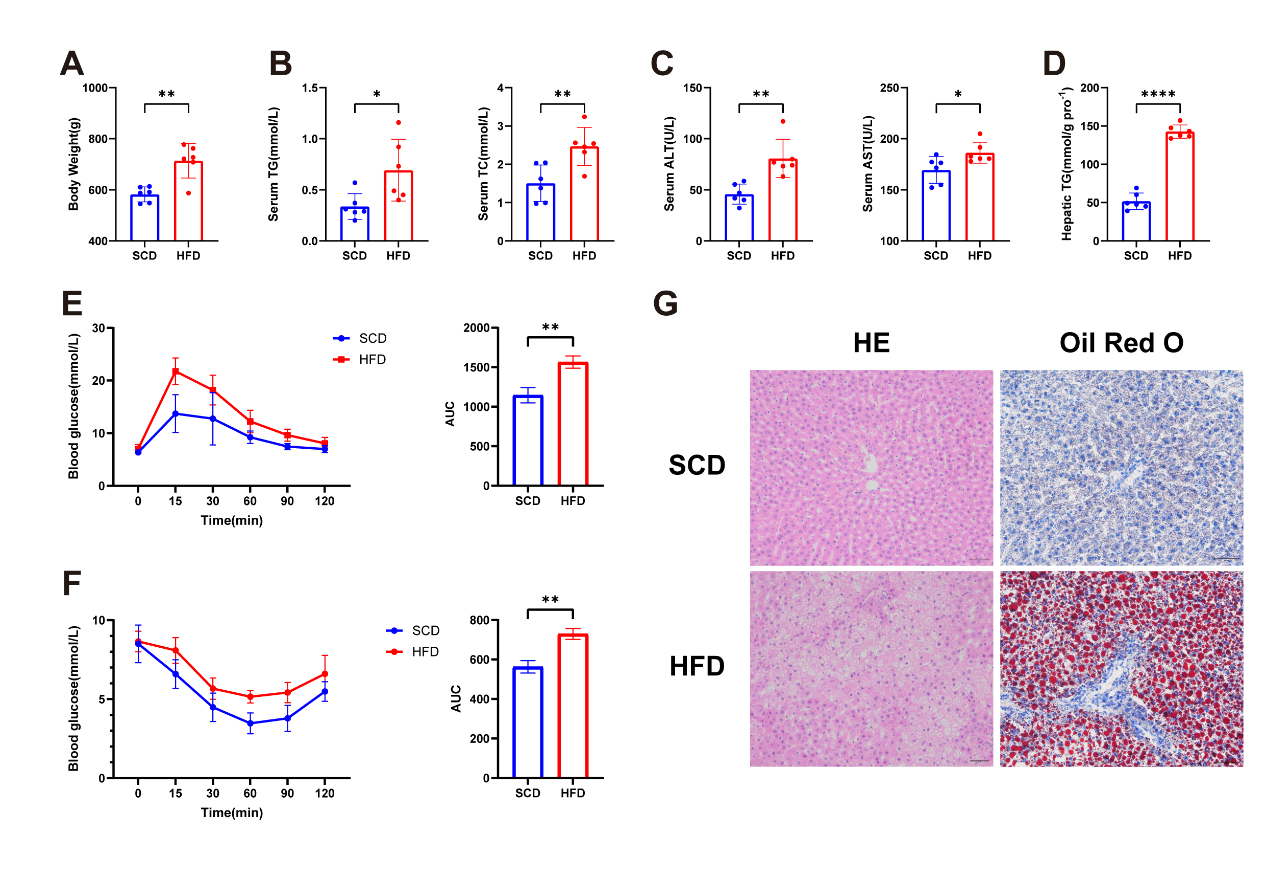
**

**Figure S1. Establishment and characterization of the HFD-induced MASLD rat model. (A)** Body weight. **(B)** Serum TC and TG levels. **(C)** Serum AST and ALT levels. **(D)** Liver TG contents in the SCD and HFD groups. **(E)** GTTs with the corresponding AUCs in the SCD and HFD groups. **(F)** ITTs with the corresponding AUCs in the SCD and HFD groups. **(G)** Representative images of H&E staining and Oil Red O staining of rat liver tissues from the SCD and HFD groups. *n* = 6 individuals/group. Each point represents an individual rat. The data are presented as the means ± SDs. **P* < 0.05, ***P* < 0.01, ****P* < 0.001, *****P* < 0.0001. The data were analyzed via unpaired Student's *t* test.

**
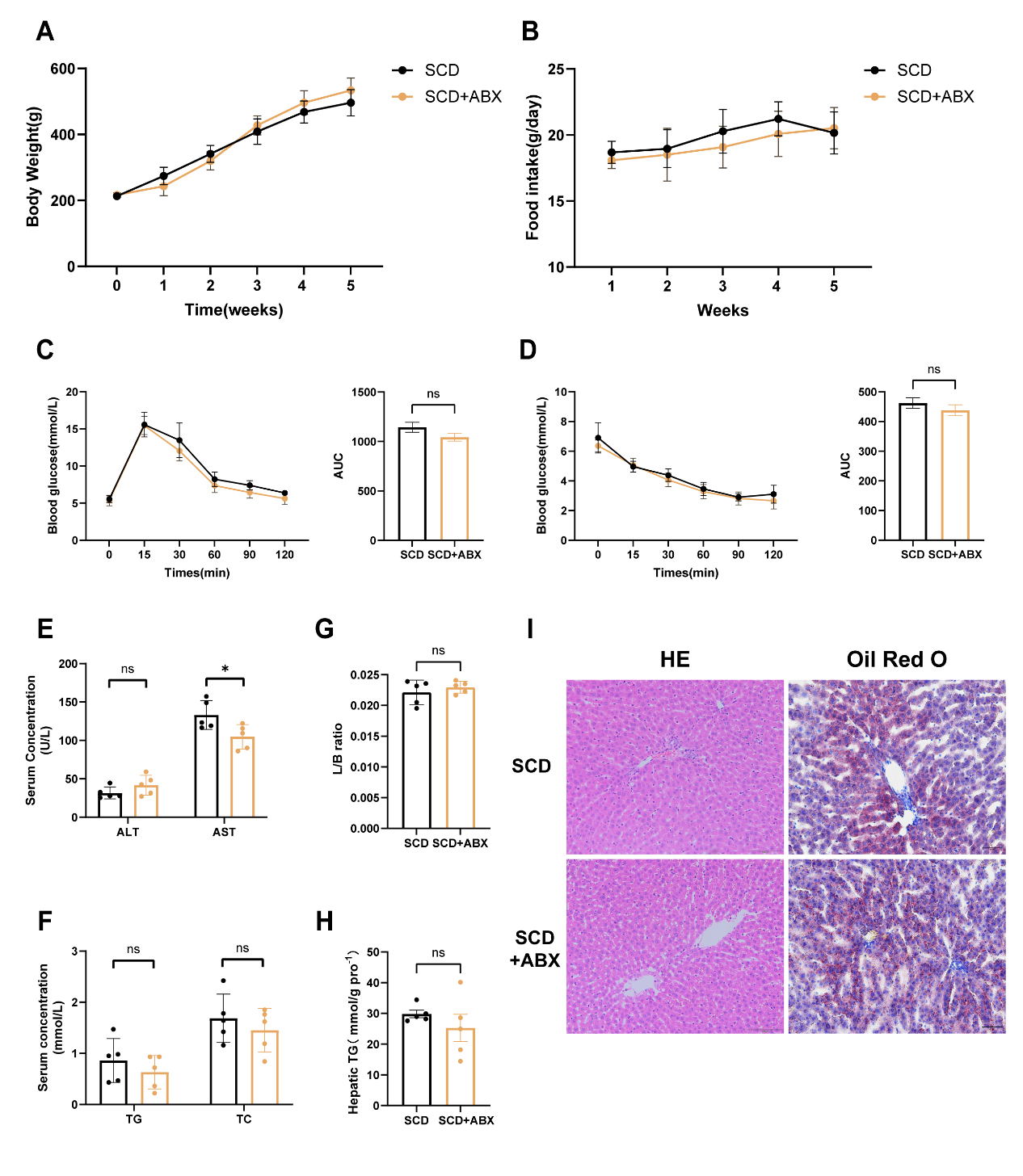
**

**Figure S2. Effects of Abx administration on SCD-fed rats.** **(A)** Body weight, **(B)** food intake. **(C)** Glucose tolerance test (GTT) with the corresponding area under the curve (AUC). **(D)** Insulin tolerance test (ITT) with the corresponding AUCs. **(E)** Serum AST and ALT levels. **(F)** Serum TC and TG levels. **(G)** Liver/body weight ratios and **(H)** liver TG contents. **(I)** Representative images of H&E staining and Oil Red O staining of rat liver tissues. *n* = 5 individuals/group. Each point represents an individual rat. The data are presented as the means±SDs. **P* < 0.05, ***P* < 0.01, ****P* < 0.001, *****P* < 0.0001. The data were analyzed via the Mann‒Whitney U test.

**
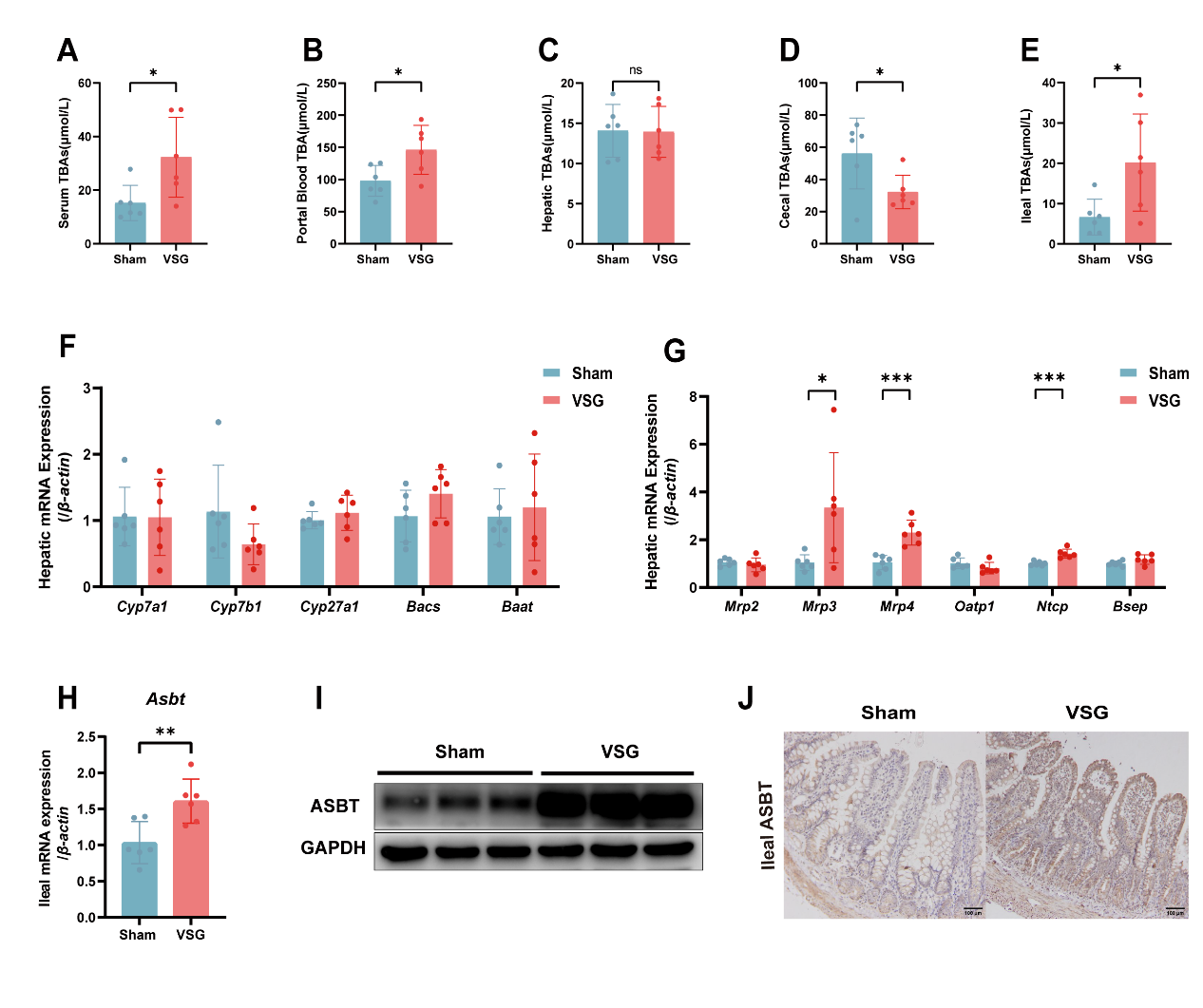
**

**Figure S3. VSG altered bile acid enterohepatic circulation and increased the reabsorption of bile acids in the ileum.** **(A-E)** The levels of bile acids in the **(A)** serum, **(B)** portal vein, **(C)** liver, **(D)** cecal contents, and **(E)** ileum. **(F)** Relative hepatic mRNA expression of genes related to bile acid synthesis (*Cyp7a1, Cyp7b1, Cyp27a1, Bacs, Baat*)**. (G)** Relative hepatic mRNA expression of genes related to bile acid transportation (*Mrp2, Mrp3, Mrp4, Oatp1, Ntcp, and Bsep*)**. (H)** Relative ileal mRNA expression of *Asbt*. **(I)** Relative ileal protein expression of ASBT. **(J)** Immunohistochemical staining of the ASBT in the ileum of the VSG and Sham groups. n = 6 individuals/group. Each point represents an individual rat. The data are presented as the means ± SDs. **P* < 0.05, ***P* < 0.01, ****P* < 0.001, *****P* < 0.0001. The data were analyzed via unpaired Student's t test.

| **Table S1. Primers for qRT‒PCR analysis.** | | |
| --- | --- | --- |
| Gene name | Forward primer sequences | Reverse primer sequences |
| *Fxr* | CAGCAGACCCTCCTGGATTA | TCTTCGTGGTCCAGTGTCTG |
| *Shp* | GAGTCTTTCTGGAGCCTTGAG | AGGACTTCACACAATGCCC |
| *Acc1* | GCCTCCAACCTCAACCACTA | AAGGTCCGGAAAGAGACCAT |
| *Fasn* | CTCTGGTGGTGTCTACATTTC | GAGCTCTTTCTGCAGGATAG |
| *Scd1* | TAGGGGAAGGCGTGATGGTAG | CTGGTACTGCTGGGGCGAAAC |
| *Cpt1α* | GATGTGGACCTGCATTCCTT | TCCTTGTAATGTGCGAGCTG |
| *G6pc* | GACCTCAGGAACGCCTTCTATG | AGGAGATTGATGCCCACAGTCT |
| *Gck* | AGATGCTATCAAGAGGAGAG | ACAATCATGCCGACCTCACAT |
| *Irs1* | CTGCTTCTGCTTCTGTTACACCTC | GGTTATGGTTGGGACTTAGGTTCA |
| *Irs2* | CTCTTTGCCCCGCTCTTACA | GGAAGGCACTGCTGAGTGAT |
| *Insr* | GCCATCCCGAAAGCGAAGATC | TCTGGGGAGTCCTGATTGCAT |
| *Fgf19* | GCCATCAAGGACGTCAGCA | CTTCCTCCGAGTAGCGAATCAG |
| *Fgfr4* | CCTTCCACGGAGAGAATCGTATC | CTACCCAGAGAGTTCTCCACAAGAC |
| *Tgr5* | GTGCTTCGAGGAAGACCCAA | AGTCCAAGTCAGTGCTGCAT |
| *β-actin* | GGTCAGGTCATCACTATCGGCAATG | CAGCACTGTGTTGGCATAGAGGTC |
